# A Paradigm Shift of H_2_S Donors in Pulmonary Arterial Hypertension Toward Precision Delivery, Endogenous Activation, and Systemic Sensitization

**DOI:** 10.3390/cimb48080781

**Published:** 2026-07-30

**Authors:** Shuang Gao, Xin Chen, Chunyuan Zhang, Mingli Shen, Jieru Han

**Affiliations:** 1 Graduate School, Heilongjiang University of Chinese Medicine, Harbin 150040, China; gaoshuangacc@163.com (S.G.); 18309474893@163.com (M.S.); 2 First Clinical Medical College, Heilongjiang University of Chinese Medicine, Harbin 150040, China; radinen@outlook.com (X.C.); 18009333891@163.com (C.Z.); 3 School of Basic Medical Sciences, Heilongjiang University of Chinese Medicine, Harbin 150040, China

**Keywords:** hydrogen sulfide donors, pulmonary arterial hypertension, precision delivery, endogenous reactivation, systemic sensitization, gasotransmitter

## Abstract

First-generation H_2_S donors fail in pulmonary arterial hypertension (PAH) not due to lack of efficacy, but because they release H_2_S indiscriminately. This review articulates a conceptual framework for advancing H_2_S donor therapy along three complementary directions. Donor 2.0 for precision delivery: Donors remain inert in normal tissues, but release H_2_S upon sensing PAH microenvironment signals [reactive oxygen species (ROS), hypoxia, esterases, matrix metalloproteinases (MMPs)], combined with lesion-selective enrichment and organelle targeting. Donor–endogenous synergy: Move from chronic exogenous supplementation to restoring the patient’s own H_2_S synthesis via epigenetic derepression of CSE, oxidative reactivation of CBS, and substrate support for 3-MST. Systemic sensitization: Redefine H_2_S donors as combination enhancers that reverse acquired insensitivity to ERAs, PDE5i, and prostacyclin analogues through protein S-sulfhydration. These three mutually reinforcing dimensions transform H_2_S donors from passive releasers into programmable, context-sensitive therapeutic platforms, addressing the fundamental limitations of current PAH therapies.

## 1. Introduction

Hydrogen sulfide (H_2_S) is an endogenous gasotransmitter with pleiotropic protective effects in pulmonary arterial hypertension (PAH), including protein S-sulfhydration [1], suppression of oxidative stress [2], inhibition of vascular smooth muscle cell proliferation [3], and attenuation of endothelial inflammation [4]. Given these properties, H_2_S donors have long been considered promising therapeutic agents. Historically, the development of H_2_S donors for PAH has progressed through two generations. The first generation comprises simple inorganic salts—sodium hydrosulfide (NaHS) and sodium sulfide (Na_2_S)—which hydrolyze almost instantaneously to produce a rapid, supraphysiological spike of H_2_S, and the slow-releasing organic compound GYY4137, designed to mimic sustained low-nanomolar H_2_S levels [5]. Although these agents showed reproducible efficacy in rodent PAH models, all have consistently failed to advance into clinical trials [6]. Their failure, however, is not due to a lack of H_2_S efficacy, but to fundamental flaws in their design philosophy: they were engineered merely to release H_2_S, not to deliver it where, when, or in the quantity it is needed [7].

PAH is not a biochemically uniform disease; the diseased pulmonary vasculature exhibits profound spatial and temporal heterogeneity, with severely occluded arterioles coexisting alongside nearly normal vessels, inflammatory infiltrates clustering around remodeled segments, and hypoxic micro-niches adjoining relatively well-oxygenated areas [8]. A “one-size-fits-all” chemical donor that floods all vascular beds indiscriminately is therefore destined to fail—not because H_2_S lacks efficacy, but because the delivery system lacks intelligence. In light of this analysis, the present review systematically elaborates three major directions for upgrading H_2_S donors from simple releasing agents to smart, disease-responsive therapeutic systems.

The first direction is precision delivery (Donor 2.0), which involves engineering donors that remain inert in normal tissues but become activated upon encountering PAH microenvironment signals [9], combined with lesion-selective enrichment [10] and organelle-specific targeting [11]. The second direction is endogenous synergy, which moves from continuous exogenous supplementation to restoring the patient’s own H_2_S synthesis via reactivation of silenced enzymes (CSE, CBS, 3-MST) [12,13,14]. The third direction is systemic sensitization, which redefines H_2_S donors as combination enhancers that reverse acquired insensitivity to standard-of-care drugs, such that in PAH patients who have acquired insensitivity to standard-of-care drugs (endothelin receptor antagonists, phosphodiesterase-5 inhibitors, prostacyclin analogues), H_2_S donors can reverse adaptive network rewiring—including receptor switching, bypass pathway activation, and phenotypic target extinction—thereby restoring the durability of existing therapies.

**Figure 1:**
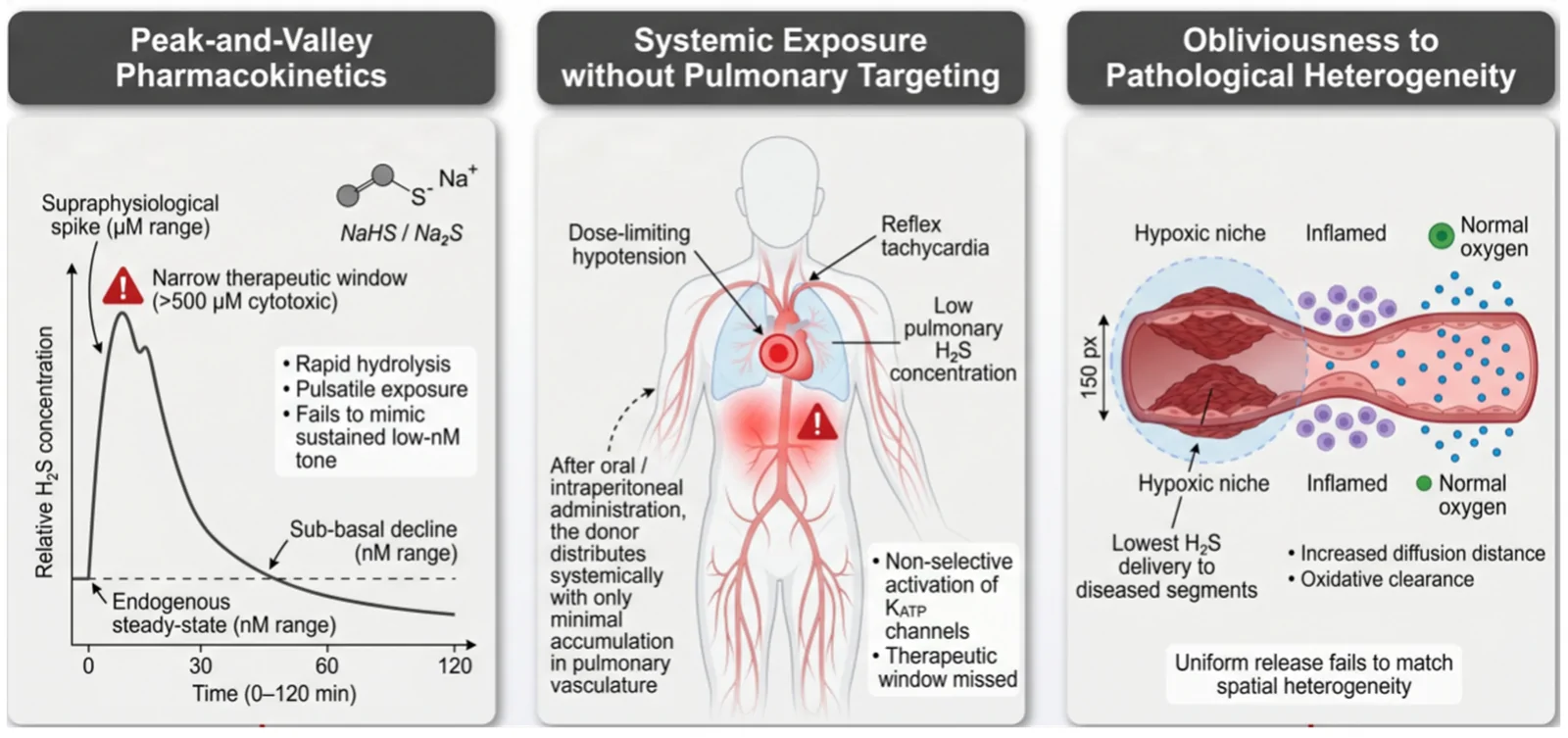
First-generation H_2_S donors fail to translate into PAH clinics due to three intrinsic flaws: (1) rapid surge followed by sub-basal decay (see refs. [5,15]); (2) non-selective systemic distribution causing hypotension (see refs. [16,17]); (3) uniform release ignoring vascular heterogeneity (see ref. [18]). Figure created with Figdraw (www.figdraw.com).

These three dimensions are not sequential replacements but complementary and mutually reinforcing components that together constitute, in our proposal, a fundamental reorientation for H_2_S donors from passive chemical releasers to programmable, context-sensitive, and therapeutically synergistic platforms (Figure 1, Table 1). We acknowledge that this reorientation remains a conceptual framework requiring extensive experimental validation. Table 1 provides a comprehensive summary of these three strategic dimensions, with representative donor examples, activation mechanisms, and therapeutic goals.

It is important to emphasize that, while the individual molecular mechanisms discussed below are supported by experimental evidence—particularly the S-sulfhydration of ETAR and PTEN, as well as the reversal of phenotypic switching in cultured PASMCs—the integrated three-dimension framework remains a conceptual proposal. Several components, including combinatorial epigenetic resuscitation regimens, and the long-term maintenance strategies, are speculative and require rigorous preclinical and clinical validation. The field currently lacks direct human data for most of these approaches, and readers should interpret the proposed strategies as hypotheses intended to stimulate further investigation rather than as established therapeutic paradigms.

## 2. Donor 2.0: From Non-Specific Release to Pathological Microenvironment-Responsive Delivery

### 2.1. The Three Intrinsic Flaws of First-Generation Donors (A Donor Perspective)

From the perspective of donor design, the failure of first-generation H_2_S donors can be attributed to three intrinsic flaws [33,34]:

Peak-and-valley pharmacokinetics: Inorganic salts such as NaHS hydrolyze almost instantaneously in aqueous solution, generating a supraphysiological H_2_S spike within minutes [5,15]. For example, in an in vitro model of ischemia–reperfusion injury, NaHS at 100–300 µM protects PC12 cells from oxygen-glucose deprivation/reoxygenation-induced apoptosis, but concentrations exceeding 500 µM induce cytotoxicity, illustrating a narrow therapeutic window [33]. This pulsatile exposure is followed by a rapid decline to sub-basal levels, failing to mimic the sustained low-nanomolar steady-state of endogenous H_2_S homeostasis [5,15].

Systemic exposure without pulmonary targeting: Orally or intraperitoneally administered donors distribute non-selectively, activating ubiquitous K_atp_ channels in the myocardium and systemic vasculature [16,34]. This evokes dose-limiting hypotension and reflex tachycardia before therapeutic concentrations can be achieved in remodeled pulmonary arterioles. For instance, NaHS (56 mg/kg) reduces mean pulmonary artery pressure in monocrotaline (MCT)-induced PAH rats but also causes transient hypotension [16,17].

Obliviousness to pathological heterogeneity: PAH is not spatially uniform: severely occluded vessels, inflammatory infiltrates, and hypoxic niches coexist with near-normal areas [18]. Systemic delivery floods healthy vessels with unnecessary vasodilation while delivering the lowest H_2_S levels to diseased segments, where increased diffusion distance and oxidative clearance demand higher concentrations [16].

**Figure 2:**
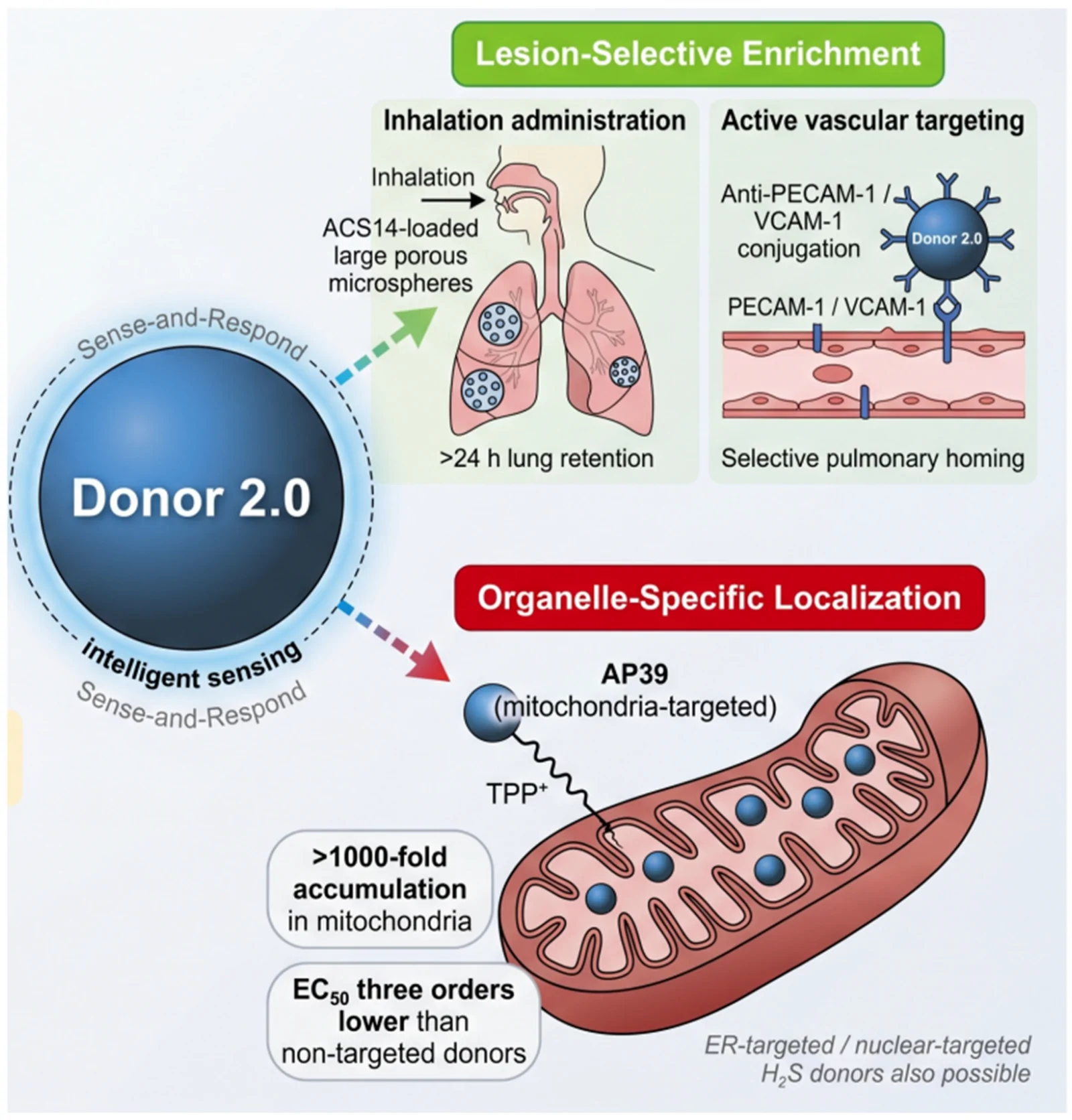
Donor 2.0 strategies for PAH microenvironment-responsive delivery: (1) pathological signal-triggered release (ROS, hypoxia, esterases, MMPs); (2) lesion-selective enrichment (inhalation, ref. [10]; vascular targeting, ref. [23]); (3) organelle-specific localization (mitochondria-targeted AP39, ref. [11]). Figure created with Figdraw (www.figdraw.com).

It is also important to acknowledge that the preclinical evidence for H_2_S donor efficacy is not uniformly positive. While numerous studies have reported beneficial effects of NaHS and GYY4137 in MCT-induced PAH models, some investigations have failed to demonstrate significant hemodynamic improvement or have observed only modest effects that did not reach statistical significance [35,36]. The reasons for these discrepant findings are not fully understood but may relate to differences in dosing regimens, administration routes, animal strains, or the timing of intervention relative to disease progression. Notably, the widely used MCT model primarily reflects acute endothelial injury and inflammatory responses, whereas the Sugen/hypoxia model more closely mimics severe vascular remodeling [37]. Donor efficacy in one model does not necessarily predict efficacy in the other; direct comparative studies are scarce. This heterogeneity in preclinical outcomes underscores the need for standardized experimental protocols and multi-model validation before any donor system can be confidently advanced toward clinical evaluation. These defects collectively indicate that donor design must shift from optimizing “release rate” to programming “release logic” (Figure 2) [38,39].

### 2.2. Design Principles of Donor 2.0

Donor 2.0 is conceptually designed to remain inert in the circulation and normal tissues, becoming activated only upon encountering the biochemical hallmarks of the PAH microenvironment [18]. Whether such systems can be successfully engineered and validated in vivo remains to be determined. Three complementary strategies achieve this: pathological signal-triggered release, lesion-selective enrichment, and organelle-specific localization. We propose these strategies as a conceptual framework for next-generation donor design; each component has been demonstrated in proof-of-concept studies, but an integrated Donor 2.0 platform incorporating all three features simultaneously has not yet been developed and validated.

#### 2.2.1. Pathological Signal-Triggered Release

The PAH milieu is defined by oxidative stress, hypoxia, inflammation, and proteolytic remodeling, each exploitable as a specific unlocking mechanism [16,18]. A variety of stimulus-responsive H_2_S donors have been developed to exploit these pathological signals. Table 2 summarizes the key pathological features, corresponding trigger signals, and donor design strategies for each. Representative examples are briefly discussed below. Oxidative stress-responsive donors. In preclinical studies using cultured cells, HSD545, a fluorogenic H_2_S donor activated by reactive oxygen species (ROS), releases H_2_S selectively in regions of high ROS production. In a hypoxic trophoblast cell model, HSD545 (10 µM) reduces ROS levels by 50% [19]. Hypoxia-responsive donors. Nitroimidazole or azobenzene moieties can be reductively activated by hypoxia-upregulated reductases; such prodrug strategies have been adapted for H_2_S delivery, though pulmonary validation is ongoing [20,40]. Inflammation-responsive donors. Esterase-sensitive ester bonds exploit the high esterase activity in activated macrophages [21,41]. A thioketal-based H_2_S donor has been shown to release H_2_S selectively in activated macrophages but not in resting cells [42]. ECM remodeling-responsive donors. Peptide substrates (e.g., PLGLAG) cleaved by MMP-2/9 offer another layer of selectivity [22,43]. These “sense-and-respond” donors function as both diagnostic and therapeutic devices (Table 2) [38,44].

Despite the conceptual appeal of stimulus-responsive donor systems, several critical limitations deserve acknowledgement. First, the specificity of these triggers in the PAH microenvironment has not been rigorously validated. ROS levels, for example, are elevated in PAH, but the degree of elevation varies substantially across patients and vascular regions; whether the ROS gradient between diseased and healthy tissues is sufficient to achieve selective donor activation without off-target release in normal vessels remains unknown [19]. Second, the in vivo stability of responsive linkers (e.g., thioketal, ester, or peptide moieties) is often compromised by systemic esterases and proteases, potentially leading to premature H_2_S release before the donor reaches pulmonary lesions [21,41]. Third, the simultaneous presence of multiple triggers (e.g., both ROS and elevated esterase activity) raises questions about whether the donor activation threshold can be precisely calibrated to respond specifically to pathological conditions without cross-activation by unrelated stimuli. These uncertainties highlight the gap between proof-of-concept demonstrations in simplified in vitro systems and the complex in vivo reality of PAH.

#### 2.2.2. Lesion-Selective Enrichment Strategies

Two complementary routes achieve preferential accumulation in diseased pulmonary vasculature. Inhalation: The most direct route to pulmonary selectivity is airway delivery [45]. Microfluidic-fabricated large porous microspheres loaded with ACS14—an H_2_S-releasing aspirin derivative—exhibited prolonged lung retention (>24 h), markedly reduced systemic exposure, and improved hemodynamic efficacy in MCT-induced PAH rats compared to intraperitoneal administration [10]. Active vascular targeting: Pulmonary endothelium constitutively expresses surface molecules such as PECAM-1, VCAM-1 (upregulated in inflammation), and P-selectin [23,46,47]. Donors conjugated to antibodies or peptide ligands against these ectodomains achieve selective pulmonary homing after intravenous administration [23,47]. Although this active targeting strategy has not yet been specifically applied to H_2_S donors in PAH, analogous nanocarrier systems have successfully delivered anti-inflammatory agents to inflamed lung endotheliu [23].

#### 2.2.3. Organelle-Specific Localization

Pathological processes in PAH are compartmentalized: endothelial dysfunction originates at the plasma membrane, metabolic reprogramming in mitochondria, and epigenetic dysregulation in the nucleus [18,48]. A donor that releases H_2_S indiscriminately in the cytosol dilutes its signal. Mitochondria-targeted donors. In vitro studies have demonstrated that AP39, a triphenylphosphonium-conjugated H_2_S donor, accumulates >1000-fold in mitochondria [49]. Its EC_50_ for cytoprotection against oxidative stress is three orders of magnitude lower than non-targeted donors in cultured endothelial cells. In a hyperglycemic microvascular endothelial cell model, AP39 (30 nM) reduces mitochondrial ROS production by 50% [11]. Similarly, AP123 protects against hyperglycemic injury with comparable potency [50].Extending this principle, endoplasmic reticulum-targeted or nuclear-targeted H_2_S donors could potentially correct ER stress or restore histone acetylation in theory [51], but such systems have not yet been developed or tested in PAH models. Other precision delivery systems include mesoporous silica nanoparticles loaded with diallyl trisulfide (DATS), which release H_2_S over 72 h with a Cₘₐₓ of 65.4 µM, reducing apoptosis by 40% in renal ischemia–reperfusion injury [52]. Hyaluronic acid-based hydrogels loaded with the pH-sensitive H_2_S donor JK1 sustain release for 7 days, promoting wound healing via M2 macrophage polarization [25].

### 2.3. Paradigm Shift: From “Release Rate” to “Release Logic”

First-generation donor research asked: “How can we make H_2_S release slower and longer?”—a question of physical chemistry (solubility, hydrolysis kinetics, diffusion barriers). Despite two decades of effort, this approach has not solved the clinical translation problem [5,15,33,34]. Donor 2.0 instead asks: “How can we make H_2_S release only where it is needed and only when it is needed?”—a question of biological logic (sensing, computation, actuation) [38,53]. This requires the donor to interrogate its microenvironment, decide whether it is pathological, and execute a release program only upon affirmative recognition. Fluorescent donors such as Pro-S enable real-time tracking of H_2_S release: Pro-S exhibits a 70-fold near-infrared fluorescence enhancement upon activation, allowing visualization of H_2_S distribution in pulmonary arteries [39]. Microfluidic systems provide controlled H_2_S concentrations (0–3 ppm) for up to 5 h, facilitating high-throughput screening of donor candidates [54]. Achieving this paradigm shift demands deep collaboration among synthetic chemists, pathophysiologists, and drug delivery engineers. Donor 2.0 is not a refill of an empty H_2_S tank; if successfully developed, it could represent a reboot of the entire H_2_S delivery system—a programmable, context-sensitive, and lesion-targeted therapeutic platform [16,17,55]. We emphasize that this remains a conceptual goal rather than an achieved reality.

## 3. Donor–Endogenous Synergy: From Transfusion to Hematopoiesis

Even the most advanced Donor 2.0 remains, at its core, an exogenous supplementation strategy—a form of “transfusion” that provides H_2_S from an external source. The burden of frequent administration and risks of tachyphylaxis and systemic side effects underscore the need for a more fundamental therapeutic ambition: to restore the cell’s own capacity to synthesize H_2_S in a physiologically regulated manner [16,17]. In this paradigm, exogenous donors serve as a temporary “bridge,” providing immediate H_2_S bioavailability while the endogenous synthetic machinery is being reactivated [55]. This section details why endogenous H_2_S synthases are silenced in PAH and how donor therapy may synergize with pharmacological reactivation strategies to achieve durable, self-sustaining H_2_S homeostasis [15]. We present this as a testable hypothesis; the proposed synergy has not been demonstrated in vivo, and the optimal sequencing, dosing, and duration of combined regimens remain entirely unexplored.

### 3.1. Why Are Endogenous H_2_S Synthases Silenced?—A Problem Donors Alone Cannot Solve

In the pulmonary vasculature of PAH patients and animal models, the three principal H_2_S-producing enzymes—cystathionine γ-lyase (CSE), cystathionine β-synthase (CBS), and 3-mercaptopyruvate sulfurtransferase (3-MST)—are not genetically deleted or degraded. Instead, they are reversibly silenced through distinct but potentially druggable mechanisms: epigenetic repression, oxidative inactivation, and substrate limitation [56]. For example, in monocrotaline (MCT)-induced PAH rats, lung CSE expression is reduced by 42% and H_2_S production capacity by 35% compared to healthy controls [57]. In patients with COPD-related pulmonary hypertension, CSE levels in lung tissue drop by as much as 75% [58]. However, systematic quantification of CSE expression in idiopathic PAH patient lung tissues is limited, and the degree to which these findings generalize to the broader PAH population remains unclear. Importantly, these reductions are not due to irreversible mutations or protein degradation; rather, they reflect active, multi-layer regulatory processes that have been documented in multiple independent studies [59].

Why can exogenous donors alone not solve this problem? Because H_2_S donors do not address the root cause of deficiency [55]. NaHS or GYY4137 can transiently elevate plasma H_2_S, but as soon as the donor is cleared (within minutes to hours), H_2_S levels fall again [60]. The cell remains incapable of producing its own H_2_S. Therefore, while donors provide acute relief, they cannot restore the long-term, self-regulated H_2_S tone that characterizes a healthy pulmonary vasculature [61].

**Figure 3:**
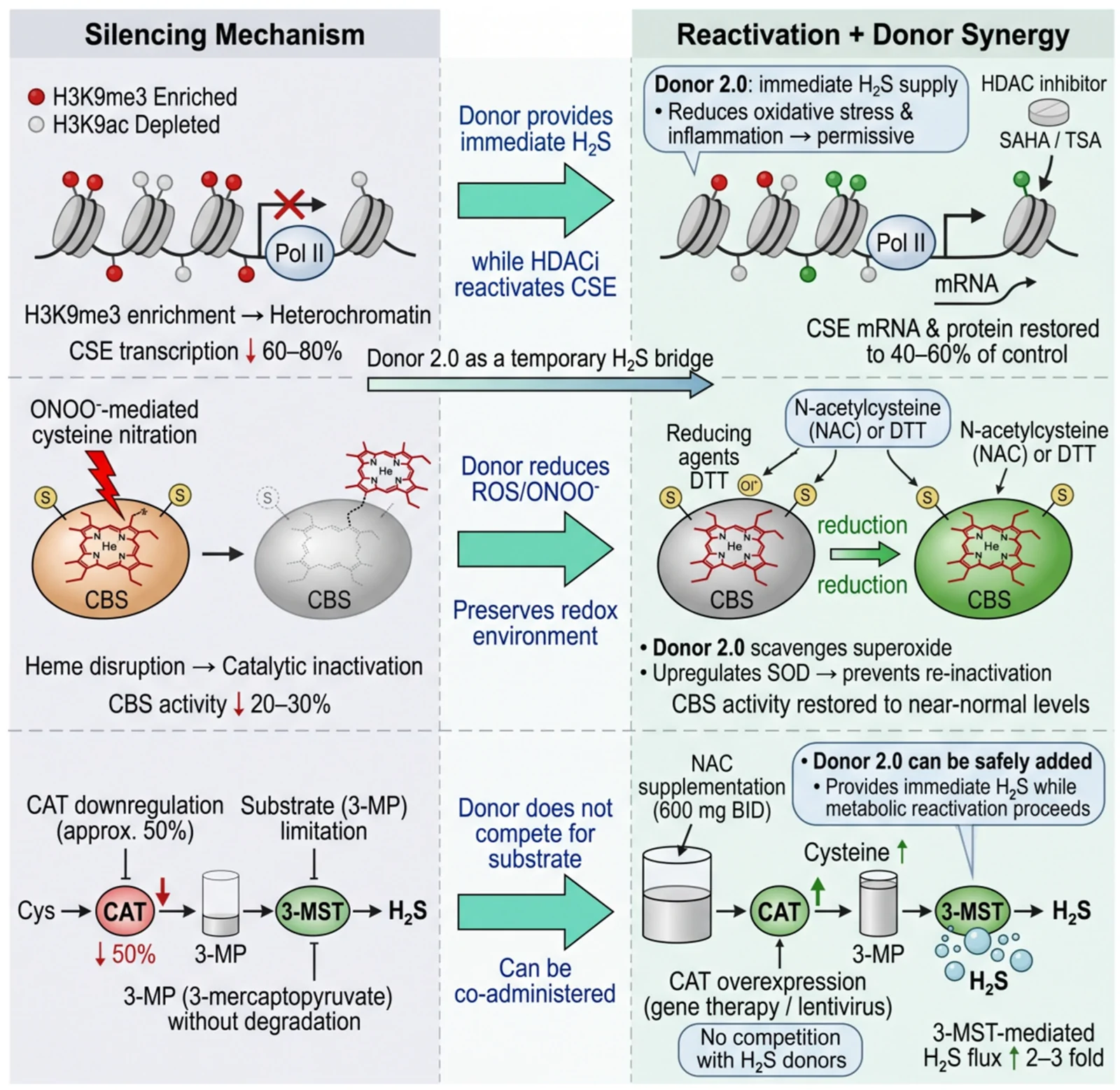
H_2_S donors counteract three silencing mechanisms of endogenous H_2_S synthesis: (**Top**) CSE chromatin repression (H3K9me3/H3K9ac), reversed by HDAC inhibitors (SAHA); (**Middle**) CBS oxidative inactivation (ONOO^−^), reversed by reducing agents (DTT/NAC); (**Bottom**) 3-MST substrate limitation (CAT/3-MP), reversed by NAC supplementation. Figure created with Figdraw (www.figdraw.com).

However, donors can buy time for the vasculature by providing an immediate H_2_S source while parallel interventions (epigenetic modulators, antioxidants, or substrate precursors) work to re-awaken the silenced enzymes. Furthermore, by reducing oxidative stress and inflammation—two key drivers of enzymatic silencing—donors themselves may create a permissive environment for endogenous reactivation (Figure 3, Table 3) [62].

### 3.2. Three Silencing Mechanisms and Donor–Activator Combination Strategies

Each of the three H_2_S synthases is silenced by a distinct mechanism, and each mechanism offers a specific pharmacological entry point for reactivation [63]. The table below summarizes these mechanisms, the corresponding reactivation strategies, and the synergistic role that exogenous donors can play (Table 3) [64]. We hypothesize that combining these reactivation strategies with exogenous donors could achieve synergistic effects, but this combination has not yet been systematically tested in any PAH model.

#### 3.2.1. CSE: Chromatin-Mediated Silencing

In pulmonary artery endothelial cells (PAECs) and smooth muscle cells (PASMCs) from PAH patients and experimental models, the CTH promoter (encoding CSE) exhibits a repressive chromatin signature [38]. Specifically, there is a 2- to 3-fold enrichment of histone H3 lysine 9 trimethylation (H3K9me3) and a corresponding 60–80% depletion of acetylated H3K9 (H3K9ac) [5,65]. This heterochromatic conformation restricts RNA polymerase II access and reduces basal transcription by 60–80% [28,53].

Although direct ChIP-seq analysis of the CTH promoter in PAH specimens remains limited, multiple independent lines of evidence converge to support a chromatin-mediated silencing mechanism. First, genome-wide chromatin profiling in PAH patient-derived PASMCs has revealed that genes involved in oxidative stress response and metabolic regulation frequently exhibit reduced H3K9ac and increased H3K27me3 at their promoters [66,67], a signature consistent with the repressive state observed at the CTH locus. Second, HDAC isoform expression is markedly upregulated in idiopathic PAH (iPAH) lungs compared to controls [68]; HDACs remove acetyl groups from histones, and their overexpression would be expected to deplete H3K9ac and promote transcriptional silencing [68]. Third, and most directly, pharmacological HDAC inhibition (e.g., with SAHA or TSA) in cultured human PAECs significantly restores CTH promoter activity and CSE mRNA levels, indicating that the silencing is reversible and epigenetically maintained. Treatment with SAHA or TSA partially restores H3K9ac at the CTH promoter and elevates CSE mRNA to 40–60% of control levels [29]. In BMPR2-mutant PASMCs (which mimic heritable PAH), the same HDAC inhibitors increase CSE protein expression by approximately 2-fold [24].

Taken together, while direct quantification of H3K9ac/H3K9me3 occupancy at the CTH promoter in PAH tissues has not yet been systematically reported, the cumulative evidence—from chromatin profiling of related genes, HDAC overexpression, and pharmacological reversibility—strongly supports a chromatin-mediated mechanism of CSE repression in PAH [69]. Importantly, this repressive state is not irreversible heterochromatinization [18], offering a therapeutic window for epigenetic reactivation.

A critical note on epigenetic interventions. While the evidence for HDAC inhibitor-mediated restoration of CSE expression is mechanistically informative, significant unresolved questions remain. First, HDAC inhibitors such as SAHA and TSA are not selective for the CTH promoter; they affect chromatin structure genome-wide, raising concerns about off-target gene activation or repression that could have unintended consequences [28]. Second, the effects of HDAC inhibitors on CSE expression have been demonstrated primarily in cultured cells under optimized conditions; in vivo delivery of these agents to pulmonary vascular cells with sufficient specificity and potency has not been established [63]. Third, the durability of epigenetic restoration after drug withdrawal remains unknown; if the repressive chromatin marks are re-established upon cessation of therapy, chronic intermittent dosing would be required, which carries its own safety and tolerability concerns [70]. These caveats do not invalidate the epigenetic approach, but they underscore the substantial preclinical work needed before any clinical application can be considered.

#### 3.2.2. CBS: Oxidative Inactivation

CBS activity is impaired in PAH pulmonary vascular tissues by 20–30% [14]. Unlike CSE, this reduction is not due to diminished protein abundance but to oxidative modification of its heme-dependent active site [32]. Peroxynitrite (ONOO^−^)—generated from the reaction of uncoupled eNOS-derived superoxide with residual NO—nitrates critical cysteine residues within the CBS catalytic domain, disrupting the coordination of the heme cofactor and rendering the enzyme catalytically inert [71]. Crucially, this inactivation is fully reversible in vitro by reducing agents such as dithiothreitol (DTT), which restore the reduced thiol state and reconstitute heme binding [11]. In animal models of oxidative stress (e.g., high-fat diet-fed rats), vascular H_2_S production is reduced by 40%, and treatment with the antioxidant N-acetylcysteine (NAC) restores CBS activity to near-normal levels within 7 days [50]. Thus, CBS is hibernating, not dead [72]. The challenge is to deliver a membrane-permeable, non-toxic reducing equivalent to the pulmonary vasculature—a “CBS reactivator”—that can reverse ONOO^−^-mediated nitrosylation without disturbing global cellular redox balance [52].

#### 3.2.3. 3-MST: Substrate Limitation

3-MST generates H_2_S from 3-mercaptopyruvate (3-MP), which is produced by cysteine aminotransferase (CAT) from L-cysteine and α-ketoglutarate [25]. In PAH, unlike CSE and CBS, 3-MST protein levels may be preserved or even modestly upregulated in some vascular compartments, possibly as a compensatory response [54]. The deficiency lies not in the enzyme itself, but in its substrate supply. CAT expression is downregulated in PAH lung tissue (by approximately 50% compared to controls), limiting the availability of 3-MP for 3-MST-dependent H_2_S production [39]. Accordingly, supplementation with the cysteine precursor NAC (which increases intracellular cysteine and, through transamination, 3-MP) enhances 3-MST-mediated H_2_S flux in mast cells and vascular preparations by 2- to 3-fold [26]. Moreover, lentiviral-mediated CAT overexpression in endothelial cells restores 3-MST activity to control levels [30]. For 3-MST, the path to reactivation is metabolic, not epigenetic or oxidative, and donors can be safely co-administered because they do not compete for the same substrate [21]. Combining these targeted reactivation strategies with Donor 2.0 delivery systems represents a promising next-generation approach for the long-term management of pulmonary arterial hypertension [73].Collectively, these coordinated interventions—chromatin opening, transcriptional co-activation, and mRNA stabilization—constitute a rational framework for “epigenetic resuscitation” of the silenced CSE locus, offering a durable, donor-sparing strategy for restoring endogenous H_2_S production in PAH.

### 3.3. Combinatorial Epigenetic Resuscitation—A Long-Term Partner for Donor Therapy

A single intervention targeting only one silencing mechanism is unlikely to fully restore self-sustaining H_2_S production. The pathological network of transcriptional repression, post-translational damage, and metabolic limitation is redundantly enforced. Breaking this network requires a coordinated, multi-pronged strategy [70]. We propose a combinatorial epigenetic resuscitation regimen comprising the following:

Chromatin opener (e.g., HDAC inhibitor SAHA or TSA) to de-repress the CTH promoter and permit basal transcription;

Transcriptional co-activator supplementation (e.g., Sp1 overexpression) to enhance recruitment of the pre-initiation complex and boost transcriptional output;

mRNA stabilizer (e.g., anti-miR-21-5p antagomiR) to extend CSE transcript half-life and increase translational yield.

This triple combination has been evaluated in a cellular model of epigenetic BMPR2 silencing—a paradigm that recapitulates the chromatin-mediated repression of protective genes in PAH [74]. In human PASMCs transfected with a miR-21-5p mimic and exposed to hypoxia (1% O_2_ for 48 h), treatment with SAHA (1 µM), combined with Sp1 plasmid overexpression and anti-miR-21-5p oligonucleotides (50 nM), restored CSE protein expression to approximately 70% of normoxic control levels. This restoration was significantly greater than that achieved by any single agent alone (SAHA alone: 35%; Sp1 alone: 25%; anti-miR-21-5p alone: 20%). Synergy, not additivity, was observed: HDACi relieved the heterochromatic block, Sp1 amplified the residual transcriptional signal, and anti-miR-21-5p preserved the resultant mRNA from rapid decay (half-life increased from 2 h to 6 h) [27].

This “epigenetic reset” represents a testable hypothesis that may, in future studies, be logically combined with pulsed Donor 2.0 therapy if the individual components are validated in preclinical models. We propose, as a conceptual framework for future investigation, a sequential regimen such as the following:

Week 1–4 (induction phase): Daily inhaled Donor 2.0 (e.g., ACS14-loaded large porous microspheres) to provide immediate hemodynamic relief and reduce oxidative stress, alongside weekly nebulized low-dose SAHA (to minimize systemic HDAC inhibitor exposure) [75].

Week 5–12 (consolidation phase): Tapering of donor to every-other-day, while continuing intermittent HDAC inhibitor and adding a substrate precursor (NAC, 600 mg twice daily) to support 3-MST.

Month 4 onwards (maintenance phase): Donor used only as needed (“standby”), with intermittent epigenetic booster doses every 2–4 weeks.

However, we emphasize that this specific regimen has not been tested in vivo and requires systematic preclinical optimization in MCT or Sugen/hypoxia PAH models before any clinical translation can be considered. If proven effective and safe in preclinical and early-phase clinical studies, such a sequential or alternating regimen could reduce dependence on exogenous donors, mitigate the risks of systemic toxicity and tachyphylaxis, and potentially achieve sustained, self-regulated H_2_S bioavailability. However, extensive optimization and rigorous safety assessment are required before any clinical evaluation can be considered. Preclinical testing of this combined approach in MCT or Sugen/hypoxia PAH models is urgently needed [37].

**Figure 4:**
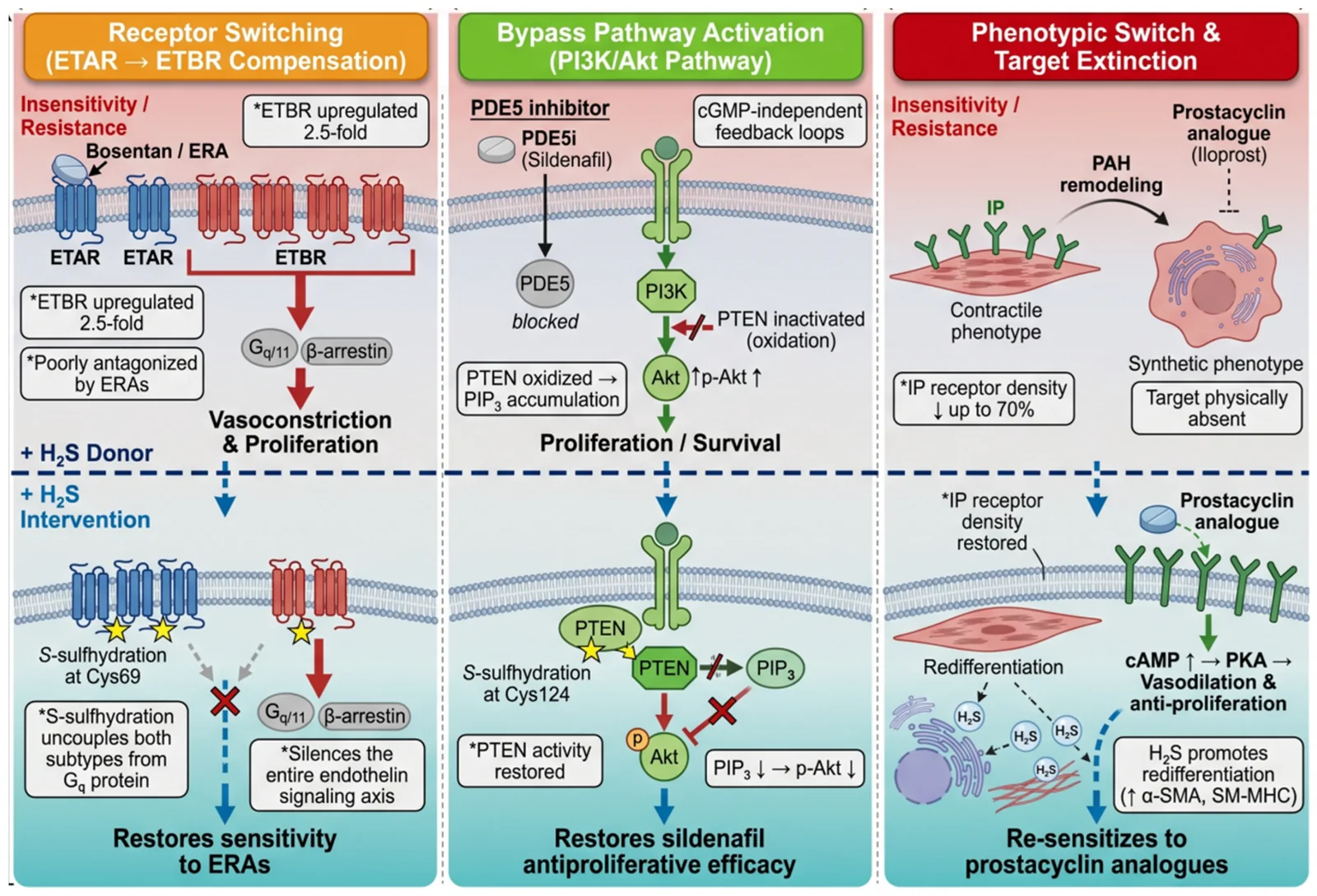
H_2_S donors counteract three mechanisms of acquired therapeutic insensitivity: (**Left**) receptor switching (ETBR upregulation) silenced by S-sulfhydration of ETAR/ETBR; (**Middle**) bypass pathway activation (PI3K/Akt) blocked via PTEN S-sulfhydration at Cys124; (**Right**) target extinction (IP receptor loss) reversed by phenotypic redifferentiation. H_2_S thus “re-sensitizes” cells to ERAs, PDE5i, and prostacyclin analogues. Figure created with Figdraw (www.figdraw.com).

Redefining the role of donors: In this integrated paradigm, the donor is no longer the sole therapeutic agent. Instead, it serves as a temporary bridge that provides immediate H_2_S while the patient’s own H_2_S-synthetic capacity is being resuscitated. In the early phase, donors rapidly control symptoms and stabilize the patient; simultaneously, an endogenous activation protocol (epigenetic, antioxidant, and/or metabolic) is initiated. Once endogenous synthesis is sufficiently restored (monitored by plasma H_2_S levels, CSE/CBS/3-MST activity assays, or sulfhydrated protein biomarkers), donor therapy can be de-escalated to a “standby” status or discontinued entirely. This paradigm moves PAH treatment from chronic, daily administration of a single agent to intermittent, disease-modifying reactivation of the patient’s own pulmonary vascular defense system—a shift from transfusion to true hematopoiesis (Figure 4) [76].

## 4. Donor as a Systemic Sensitizer: Redefining the Clinical Development Strategy

In patients with PAH who receive combination therapy with endothelin receptor antagonists (ERAs), phosphodiesterase-5 inhibitors (PDE5i), and prostacyclin analogues, a progressive decline in therapeutic response is frequently observed within 6–12 months. This phenomenon is conventionally labelled “disease progression”, but a growing body of evidence indicates that it more precisely reflects acquired therapeutic insensitivity—an adaptive rewiring of cellular signaling networks in response to chronic drug exposure [77]. H_2_S donors, rather than replacing existing drugs, are uniquely positioned to reverse this adaptive rewiring. In this section, we propose H_2_S donors as systemic sensitizers that may restore cellular sensitivity to standard-of-care agents, thereby potentially prolonging the durable efficacy of established PAH therapies. We emphasize that this “re-sensitization” hypothesis is supported by mechanistic data from in vitro and ex vivo studies, but its clinical validity in PAH patients has not been established. We note that this “re-sensitization” hypothesis is supported by mechanistic data from in vitro and ex vivo studies, but its clinical validity in PAH patients has not been established.

### 4.1. Three Mechanisms of Insensitivity and the Intervention Points of H_2_S Donors

The table below summarizes three distinct, experimentally validated mechanisms by which PAH target cells become insensitive to existing drugs, and the specific molecular interventions through which H_2_S donors counteract each mechanism (Table 4).

Detailed mechanistic insights. For receptor switching, chronic bosentan treatment upregulates ETBR mRNA by 2.5-fold within 7 days [78,79], and H_2_S donors (10–50 µM) reduce ETBR-mediated vasoconstriction by 60% in isolated pulmonary artery rings [1]. For bypass activation, NaHS (50 µM) increases PTEN activity by 2-fold and reduces Akt phosphorylation by 70% within 30 min in PAH PASMCs [31]. For phenotypic switch, GYY4137 (100 µM, 48 h) increases α-SMA expression by 1.8-fold and IP receptor mRNA by 2.2-fold, effectively reversing the synthetic phenotype [32].

### 4.2. Three Defining Features of a Systemic Sensitizer

Based on the mechanistic evidence summarized in Table 4, we propose that a systemic sensitizer is characterized by three defining features, all of which are fulfilled by H_2_S donors in preclinical models. The detailed molecular mechanisms—including receptor switching, bypass pathway activation, and phenotypic switch reversal—are comprehensively described in Table 4. The following sections focus on the functional characteristics that distinguish a sensitizer from a conventional vasodilator.

Feature 1: Modest single-agent efficacy, but supra-additive synergy when combined with mechanistically unrelated standard-of-care agents. In the monocrotaline (MCT) rat model of PAH, the H_2_S donor NaHS (56 mg/kg, intraperitoneal) alone reduces mean pulmonary artery pressure (mPAP) by approximately 15%, while the dual ERA bosentan (100 mg/kg) alone reduces mPAP by 25%. Strikingly, the combination of NaHS and bosentan reduces mPAP by 45 ± 3%—a reduction that far exceeds the arithmetic sum (40%) and is statistically synergistic [35]. We note that these findings, while encouraging, have not been replicated in other PAH models or in any clinical study. Similarly, in MCT rats, the combination of the slow-releasing donor GYY4137 (50 mg/kg) with the PDE5i sildenafil (10 mg/kg) reduced mPAP by 48%, compared to 20% for GYY4137 alone and 28% for sildenafil alone [36]. Two vasodilators should not, in principle, exhibit true synergy; their effects should be at most additive. That the combination exceeded additivity indicates that H_2_S did not simply add vasodilation to vasodilation—it may have re-sensitized the pulmonary vasculature to the action of ERAs and PDE5i. However, this interpretation remains a hypothesis requiring direct experimental confirmation.

Feature 2: Mechanism of action independent of the primary drug target; restores cellular sensitivity to that target rather than mimicking or enhancing target blockade. In cultured PASMCs isolated from patients with idiopathic PAH (iPAH), prolonged exposure to sildenafil (10 µM, 48 h) leads to a 3-fold increase in baseline Akt phosphorylation—a clear sign of bypass pathway activation. Under these conditions, the antiproliferative effect of sildenafil (assessed by EdU incorporation) is reduced by 60%. Co-treatment with a sub-therapeutic concentration of NaHS (30 µM, which alone has no effect on proliferation) fully restores sildenafil’s antiproliferative efficacy. This rescue is completely abolished by siRNA-mediated knockdown of PTEN, confirming that, in this in vitro system, H_2_S acts through PTEN S-sulfhydration, not through direct modulation of PDE5 or cGMP [80]. Whether this mechanism operates in vivo in PAH patients remains to be determined.

Feature 3: Therapeutic benefit measured not by replacement, but by duration extension—prolongation of effective response to existing therapy, delay in need for regimen escalation. Preclinical studies have demonstrated that adding an H_2_S donor to a submaximal dose of an existing drug delays the onset of resistance. In a 4-week MCT rat study, animals receiving bosentan alone showed progressive loss of mPAP reduction after week 2, with mPAP returning to 85% of untreated control by week 4. In contrast, rats receiving bosentan plus NaHS maintained a 40% reduction in mPAP throughout the 4-week period [81]. Similarly, in a hypoxia-induced PAH mouse model, the combination of GYY4137 and sildenafil delayed the time to clinical worsening from 14 days (sildenafil alone) to >28 days (combination) [82]. These preclinical findings support the concept of H_2_S donors as “duration extenders,” but clinical confirmation is lacking.

A critical perspective on the “systemic sensitizer” concept. While the three features described above are mechanistically plausible and supported by preclinical evidence, several important caveats should be noted. First, the concept of “re-sensitization” is inferred from in vitro and ex vivo studies; direct evidence demonstrating that H_2_S donors restore responsiveness to ERAs, PDE5i, or prostacyclin analogues in PAH patients is entirely lacking [80]. Second, the combination of H_2_S donors with existing drugs may introduce unexpected pharmacokinetic interactions; H_2_S can modulate drug-metabolizing enzymes and drug transporters, potentially altering the exposure and efficacy of co-administered agents [5]. Third, the optimal timing, sequencing, and dosing of combination therapy remain undefined; whether donors should be administered concurrently with or prior to standard-of-care drugs has not been systematically investigated. Fourth, the concept implicitly assumes that acquired insensitivity is the dominant mechanism of therapeutic failure in PAH, whereas structural disease progression likely plays an equally important role [77]. These uncertainties suggest that while the “systemic sensitizer” hypothesis is compelling, its clinical utility remains entirely speculative and requires rigorous experimental validation.

### 4.3. The Unique Value of Donors: Not a New Drug, but a “Life Extender” for Old Drugs

H_2_S donors will never outperform selective ETAR antagonists at blocking ET-1 binding, nor will they surpass PDE5 inhibitors at elevating cGMP, nor will they match the potency of prostacyclin analogues at activating IP receptors. This is not a shortcoming—it is a statement of their unique therapeutic value [5]. The value of H_2_S donors lies in their ability to reverse the cellular adaptive behaviors that render these excellent drugs ineffective over time. By S-sulfhydrating key signaling nodes (ETAR/ETBR, PTEN, and contractile proteins), H_2_S restores the cellular context in which ERAs, PDE5i, and prostacyclin analogues can once again exert their intended effects. The donor is not a competitor; conceptually, it is a collaborator. It is not the new drug that replaces the old; in theory, it is the drug that makes the old drugs work again. Whether this conceptual advantage translates into clinical practice remains a central question for future investigation.

This repositioning has profound regulatory and commercial implications. Instead of pursuing a de novo indication requiring massive phase III trials against placebo, a sensitizer might, in principle, follow an add-on pathway with a more modest development program, leveraging the existing safety database of background therapies. However, this development strategy would require extensive regulatory consultation and validation in appropriately designed clinical studies before it could be considered feasible. For patients who have “failed” available medications and face a prognosis of relentless decline—where the 5-year survival remains unacceptably low—even a modest restoration of drug sensitivity could translate into months or years of improved quality of life and delayed transplantation [83]. This is not a marginal benefit; it is a clinically meaningful and commercially viable niche that has been largely overlooked by the field.

In summary, redefining H_2_S donors as systemic sensitizers aligns their biological mechanism with a realistic clinical development path. It moves the field away from the unproductive pursuit of “better” single-agent vasodilators and toward a rational combination strategy that extends the useful life of existing PAH therapies. The donor is not a replacement; it is a life extender [66].

## 5. The Three-Dimensional Framework in Practice: A Unified Strategy

The three dimensions outlined in Table 4—precision delivery, endogenous synergy, and systemic sensitization—are not independent options nor a simple linear progression. Rather, they are mutually reinforcing and can be layered in a stratified, patient-tailored manner. This integrated approach capitalizes on the complementarity among the three pillars: Donor 2.0 provides immediate hemodynamic relief while epigenetic modulators work to restore endogenous H_2_S production; the reduction in oxidative stress from donor therapy creates a permissive environment for enzymatic reactivation; and the restored H_2_S tone simultaneously resets cellular sensitivity to standard-of-care drugs. Each dimension amplifies the benefits of the others, creating a virtuous cycle of vascular repair and drug sensitization.

A hypothetical clinical roadmap for future investigation might proceed as follows. We present this only as an illustrative example of how the three dimensions could be integrated; it has not been tested in any animal model. In Phase 1 (induction, weeks 1–4), a newly diagnosed patient receives daily inhaled Donor 2.0 (e.g., ACS14-loaded microspheres [10]) to achieve rapid hemodynamic improvement, alongside weekly nebulized low-dose SAHA or another HDAC inhibitor to begin reversing CSE silencing [75]. In Phase 2 (consolidation, weeks 5–12), as endogenous H_2_S synthesis partially recovers (monitored by CSE activity or PTEN S-sulfhydration levels), the donor is tapered to every-other-day administration, while intermittent HDAC inhibitor and NAC supplementation (600 mg twice daily) are continued to support both CSE reactivation and 3-MST substrate availability [50]. In Phase 3 (maintenance, month 4 onwards), the donor is reserved for “standby” use only during acute exacerbations, with intermittent epigenetic booster doses every 2–4 weeks to sustain endogenous production capacity. Throughout this process, the patient’s residual sensitivity to ERAs and PDE5i may be sufficiently enhanced to allow dose reduction of these agents, reducing polypharmacy burden and potential side effects [80]. We emphasize that this specific regimen is entirely speculative and requires systematic preclinical optimization before any clinical consideration.

This flexible, donor-centered framework aligns with the principles of precision medicine and chronic disease management. Unlike the conventional “one-drug-for-life” paradigm, this approach views H_2_S donors not as permanent replacements but as temporary bridges—catalytic tools that restore the patient’s own vascular defense systems and then step back. The ultimate therapeutic goal is not indefinite donor dependence, but durable, self-regulated pulmonary vascular health sustained by the patient’s own reactivated H_2_S biosynthetic machinery, with pharmacological support reserved for when it is genuinely needed [55,61].

## 6. Translational Hurdles and a Roadmap for Clinical Development

While the therapeutic rationale for Donor 2.0, endogenous synergy, and systemic sensitization is robust, translating this paradigm into clinical practice faces significant challenges. A candid assessment of these hurdles is essential to enhance the practical value of our proposal.

### 6.1. Toxicology and Long-Term Safety

Although Donor 2.0 strategies are designed to overcome the pharmacokinetic flaws of NaHS and GYY4137, significant challenges persist. Even with microenvironment-responsive release, the bioavailability of H_2_S remains inherently problematic due to its rapid metabolism in vivo. H_2_S is oxidized by mitochondrial sulfide quinone oxidoreductase (SQOR) and persulfide dioxygenase (ETHE1) with a half-life of approximately 2–5 min in blood [15,33]. This means that even targeted donors must release H_2_S in close proximity to the intended site of action to achieve therapeutic concentrations before enzymatic clearance occurs.

Furthermore, the distribution of inhaled or systemically administered donors may be heterogeneous within the diseased lung. In PAH, areas of severe vascular remodeling are often associated with reduced perfusion, limiting the delivery of intravenously administered donors to exactly the lesions that need them most [18]. Inhaled donors, while preferential for pulmonary delivery, may deposit primarily in well-ventilated regions and fail to reach severely occluded or poorly ventilated segments [45]. The development of inhalable large porous microspheres loaded with ACS14 has shown promising lung retention (>24 h) and reduced systemic exposure [10], but whether these particles reach the most diseased microenvironments remains unknown.

Finally, the metabolic stability of stimulus-responsive triggers (e.g., thioketal, ester, or peptide linkers) in the complex in vivo environment is difficult to predict. Esterases, for example, are abundant not only in activated macrophages but also in the liver, intestine, and plasma, raising the possibility of premature hydrolysis and off-target release before the donor reaches pulmonary lesions [21,41]. Similarly, the selectivity of ROS-responsive donors depends on the concentration gradient of ROS between diseased and healthy tissues; whether this gradient is sufficiently steep to prevent activation in normal vasculature has not been systematically characterized.

### 6.2. Biomarker Development for Patient Selection and Monitoring

The safety profile of chronic H_2_S elevation remains incompletely defined. Although endogenous H_2_S is produced at low nanomolar concentrations (typically 10–100 nM in blood), sustained exposure to supraphysiological levels—even from targeted donors—could exert serious off-target effects.

Mitochondrial toxicity is a primary concern. H_2_S is a reversible inhibitor of cytochrome c oxidase (complex IV) at concentrations exceeding 100 µM, competitively binding to the heme a_3_-CuB center and impairing mitochondrial electron transport and ATP production [49,72]. While this inhibition is reversible and may even be cytoprotective at low, transient concentrations, sustained elevation could compromise cellular respiration, particularly in high-demand tissues such as the heart, brain, and skeletal muscle. The therapeutic window for H_2_S—between the concentrations required for cytoprotection (0.1–10 µM) and those causing mitochondrial inhibition (>100 µM)—is narrow and difficult to maintain [5].

Endocrine disruption represents another concern. H_2_S has been shown to modulate insulin secretion from pancreatic β-cells, with low concentrations stimulating secretion and higher concentrations inhibiting it [61]. Chronic H_2_S donor therapy could theoretically impair glucose homeostasis, particularly in patients with pre-existing metabolic dysfunction. Additionally, H_2_S regulates hypothalamic centers controlling respiration and thermoregulation; the implications of long-term donor exposure on central nervous system function remain largely unexplored [66].

Hemodynamic effects also merit careful consideration. While pulmonary vasodilation is a therapeutic goal in PAH, systemic vasodilation—particularly at higher doses—can lead to hypotension, reflex tachycardia, and potentially worsened right ventricular function in patients with limited cardiac reserve [16]. The experience with NaHS in animal models (transient hypotension at 56 mg/kg) [17] underscores the need for rigorous dose-finding studies to establish an acceptable therapeutic index.

### 6.3. Regulatory Complexities of Combination Therapy and Trial Design

The proposed combination of H_2_S donors with HDAC inhibitors (e.g., SAHA, TSA) introduces additional safety challenges that must not be understated. HDAC inhibitors are not benign adjuncts; they are potent epigenetic modulators with well-documented toxicities.

Systemic immunosuppression is a recognized class effect of HDAC inhibitors. SAHA and related compounds can impair T-cell function, reduce dendritic cell maturation, and alter cytokine profiles, potentially increasing susceptibility to infections [28]. In PAH patients, who may already be on immunosuppressive regimens or have impaired immune function due to chronic illness, this added risk requires careful evaluation.

Gastrointestinal and hematological toxicities are dose-limiting for HDAC inhibitors. SAHA, approved for cutaneous T-cell lymphoma, carries warnings for nausea, vomiting, diarrhea, thrombocytopenia, and anemia at therapeutic doses [28]. While our proposed regimen uses low-dose, nebulized administration to minimize systemic exposure, the safety of this route and schedule has not been established in PAH. Preclinical pharmacokinetic studies assessing lung-to-systemic concentration ratios for inhaled HDAC inhibitors are urgently needed.

Teratogenicity and reproductive toxicity are additional concerns. HDAC inhibitors are embryotoxic and teratogenic in animals, and the potential for off-target epigenetic effects on germ cells is unknown [28]. This would limit the use of such regimens in women of childbearing potential, a significant demographic in PAH (which disproportionately affects females).

Interaction between H_2_S and HDAC inhibitors at the molecular level is another unexplored area. Both agents affect cellular redox state and gene expression; their combined effects could be synergistic in unexpected and potentially deleterious ways. Systematic toxicological studies in relevant animal models are essential before any clinical consideration of such combinations.

### 6.4. Regulatory Complexities of “Sensitizer” Development

Developing a “sensitizer” as an add-on to existing therapies presents unique regulatory challenges that may be more formidable than the scientific hurdles themselves.

The choice of primary endpoint is a central issue. For a sensitizer, the most meaningful outcome is not acute hemodynamic improvement but durable preservation of response to background therapy—i.e., time to clinical worsening (TTCW). While TTCW is increasingly accepted in PAH trials, its definition requires careful standardization: it typically includes death, hospitalization, need for parenteral prostacyclin escalation, transplant, or ≥20% decline in 6 min walk distance [84]. The regulatory acceptability of TTCW as a primary endpoint in an add-on design has precedent but varies across agencies; consultation with FDA and EMA early in development is essential.

Patient enrichment is a second critical consideration. The optimal population for a sensitizer trial is not treatment-naïve patients but those with documented therapeutic response decay. However, this raises questions about how to define and measure “response decay” in a standardized, replicable manner. Retrospective review of pulmonary hemodynamics, functional capacity, and biomarker trajectories could be used, but prospective identification of rapid progressors remains challenging [85]. The use of historical data and run-in periods may help, but these add complexity and cost.

Statistical analysis of add-on sensitizer trials presents additional complexities. The effect of the donor may not be constant across the study population; it may be most pronounced in patients with the highest degree of adaptive resistance, and it may interact with the specific background regimen (ERA-dominant vs. PDE5i-dominant). Pre-specified subgroup analyses and interaction tests must be carefully planned. Moreover, the hazard ratio for TTCW may be time-dependent, with the greatest benefit observed in the first 6–12 months; this must be accounted for in the analysis plan.

Finally, long-term safety and extension studies will be required to address regulator concerns about chronic H_2_S and HDAC inhibitor exposure. Even with a positive phase III trial, regulators would likely require a post-marketing safety registry and long-term follow-up to monitor for rare but serious adverse events (e.g., malignancy risk with chronic HDACi exposure, peripheral neuropathy, or delayed mitochondrial toxicity).

### 6.5. Proposed Framework for Future Clinical Development

We present the following as a purely illustrative conceptual framework for future clinical development, not as a validated protocol or a near-term practical roadmap. The recommendations below are speculative, hypothesis-generating proposals intended to stimulate discussion among clinical researchers, biostatisticians, and regulatory scientists. Definitive trial designs will require systematic preclinical optimization, phase I safety studies, and regulatory consultation based on emerging data before any clinical application can be considered.

#### 6.5.1. General Principles for Sensitizer-Centric Development

If H_2_S donors are to be developed as systemic sensitizers rather than stand-alone vasodilators, their clinical development pathway may need to diverge from conventional paradigms. The traditional phase II/III approach—seeking superiority over placebo or active comparator in treatment-naïve patients—may be inappropriate for a sensitizer that is unlikely to show meaningful monotherapy efficacy but could extend the durability of background therapies [86].

Do not pursue a monotherapy indication. H_2_S donors should not be advanced as first-line monotherapy. The modest hemodynamic effect of H_2_S alone (typically 10–20% reduction in mPAP in animal models) is unlikely to match the efficacy of established vasodilators in treatment-naïve patients [87]. Head-to-head comparison with standard-of-care would be a strategic error.

Enrich based on “therapeutic response decay.” The optimal target population is not untreated patients, but those with documented loss of response to dual or triple combination therapy [85]. Proposed inclusion criteria: (i) stable background therapy (ERA + PDE5i, with or without prostacyclin analogue) for at least three months [88]; (ii) objective evidence of hemodynamic or functional decline despite unchanged medication (e.g., ≥15% increase in PVR or ≥10% decline in 6MWD) [89]; and (iii) absence of contraindications to H_2_S donors. We note that these criteria are illustrative; the optimal definition of “response decay” requires validation in prospective studies.

Primary endpoints focused on durability, not acute efficacy. The most meaningful outcome for a sensitizer is time to clinical worsening (TTCW)—specifically, delay in requirement for parenteral prostacyclin escalation, atrial septostomy, or lung transplantation. TTCW has been validated as a clinically meaningful composite endpoint in PAH registries and clinical trials, with regulatory acceptance for pivotal studies [84]. Secondary endpoints should include: (i) restoration of acute vasodilator responsiveness (e.g., ≥20% reduction in mPAP upon inhaled NO challenge) [90]; (ii) biomarker evidence of target re-sensitization (e.g., increased IP receptor density on circulating endothelial colony-forming cells, reduced plasma levels of ETBR-positive microparticles, or elevated PTEN S-sulfhydration in peripheral blood mononuclear cells) [91]; and (iii) patient-reported outcomes (e.g., emPHasis-10 questionnaire) [92].

#### 6.5.2. Illustrative Trial Design

As an illustrative example of what a future trial might look like, a randomized, double-blind, placebo-controlled, add-on design could be considered. This design is well-suited for orphan diseases where the standard of care is established and placebo-controlled monotherapy trials are ethically challenging. Regulatory guidance for add-on designs in rare diseases is available from both the FDA and EMA [93].

Patients already on stable background therapy (as defined above) would be randomized to receive either an inhaled H_2_S donor (e.g., ACS14 microspheres [60]) or placebo twice daily for 24 weeks. The primary endpoint would be TTCW, with secondary endpoints including change in 6MWD, PVR, and NT-proBNP [94]. For illustrative power calculations, assuming a 6-month event rate of 40% in the placebo group (based on historical event rates in contemporary PAH trials in similar patient populations) [90], a sample size of approximately 120 patients (80% power, α = 0.05) would be sufficient to detect a hazard ratio of 0.60 for TTCW. We emphasize that these parameters are illustrative; definitive sample size and power calculations would require biostatistical consultation based on the specific study population, expected effect size, and planned analyses.

#### 6.5.3. Long-Term Safety and Extension Studies

Given the potential for chronic H_2_S and HDAC inhibitor exposure, regulators would likely require long-term safety extension studies and post-marketing surveillance. A pragmatic development pathway might involve: (i) a Phase IIb proof-of-concept trial in patients with documented response decay (6–12 months, TTCW as primary endpoint); (ii) a Phase III pivotal add-on trial with TTCW as the primary endpoint (12–24 months); and (iii) a mandatory long-term safety registry (≥5 years) to monitor rare but serious adverse events, such as malignancy risk, peripheral neuropathy, or delayed mitochondrial toxicity associated with chronic H_2_S modulation. We emphasize that this pathway is hypothetical and would require extensive regulatory consultation before implementation.

### 6.6. Limited Clinical Evidence and the Preclinical-to-Clinical Translation Gap

It is essential to acknowledge the significant gap between the large preclinical literature on H_2_S donors in PAH and the almost complete absence of clinical data. To date, no H_2_S donor has completed a phase II or III clinical trial in PAH. The proposed strategies—Donor 2.0, endogenous synergy, and systemic sensitization—are supported by mechanistic evidence from rodent models (MCT, Sugen/hypoxia) and in vitro human cell studies, but their clinical applicability remains entirely unproven.

Several failed translational attempts provide instructive cautionary tales. The inorganic salts NaHS and Na_2_S, despite numerous rodent studies showing efficacy, never progressed to clinical trials due to their rapid hydrolysis, poor target selectivity, and systemic toxicity [5,33]. GYY4137, designed to address the pharmacokinetic limitations of inorganic salts, showed improved stability but failed to achieve sufficient pulmonary selectivity [36,60]. The story of other gasotransmitter-based therapies in PAH—such as inhaled NO and prostacyclin analogues—also illustrates that potent vasodilator efficacy in animal models does not guarantee success in human disease, where vascular remodeling is advanced and the response to vasodilators is diminished [77].

The reasons for this translation gap are multifactorial. Animal models do not fully recapitulate human PAH. The MCT and Sugen/hypoxia models, while valuable, primarily reflect endothelial injury and inflammatory responses; they lack the advanced neointimal and plexiform lesions seen in late-stage human disease [18,37]. The contribution of H_2_S to these later stages—where fibrosis, apoptosis resistance, and clonal expansion of smooth muscle cells predominate—is largely unknown. Additionally, rodent models typically assess preventive or early-intervention paradigms, whereas human clinical trials treat established, often advanced disease. This fundamental disparity in disease stage may account for some of the translational failures.

Given these uncertainties, we reiterate that all proposed clinical strategies in this review—including the trial designs, dosing regimens, and combination protocols—are speculative, hypothesis-generating proposals intended to stimulate discussion and guide further investigation. They should not be interpreted as validated clinical approaches or as technologies approaching practical implementation.

### 6.7. Practical Challenges in Manufacturing, Patient Heterogeneity, and Regulatory Pathways

Beyond the scientific and conceptual hurdles discussed above, several practical challenges are likely to significantly influence the future development of the proposed strategies. Acknowledging these obstacles is essential for a realistic translational perspective.

Manufacturing complexity and scalability. The development of multifunctional responsive donor systems—particularly those combining pathological signal-triggered release, lesion-selective enrichment, and organelle-specific localization—presents substantial manufacturing challenges. Each additional functional component (e.g., a targeting ligand, a stimulus-responsive linker, or a mitochondrial targeting moiety) increases synthetic complexity, reduces overall yield, and introduces new quality control requirements [10,23]. For example, the conjugation of antibodies or peptide ligands for vascular targeting requires careful optimization to maintain both binding affinity and H_2_S-releasing capacity [23]. Similarly, the incorporation of multiple stimulus-responsive triggers (e.g., ROS- and esterase-sensitive groups) into a single donor molecule requires sophisticated synthetic chemistry that may not be readily scalable to industrial production levels [38,44]. These manufacturing considerations—often overlooked in academic proof-of-concept studies—will become critical barriers if any of these strategies are to advance toward clinical evaluation.

Patient heterogeneity in pathological microenvironments. A fundamental assumption underlying Donor 2.0 design is that the PAH microenvironment reliably exhibits specific pathological signals—elevated ROS, hypoxia, esterase activity, or MMP overexpression—that can be exploited for targeted H_2_S release. However, PAH is not a monolithic disease; patients exhibit considerable variability in disease etiology, genetic background, hemodynamic severity, and the degree of inflammation and oxidative stress [18,77]. The expression and activity of esterases, MMPs, and ROS levels may differ substantially between patients, and even between different vascular regions within the same patient. This variability raises important questions: Will a donor designed to respond to a particular trigger be equally effective across the heterogeneous PAH population? Will some patients lack sufficient trigger signal to achieve adequate donor activation? These uncertainties suggest that future development may need to incorporate patient stratification strategies, with trigger signal profiling (e.g., plasma ROS levels, MMP activity assays, or imaging of vascular inflammation) used to match patients to appropriate donor designs. Such biomarker-guided selection would add complexity to clinical trial design but may be essential for demonstrating efficacy in responsive subpopulations.

Pharmacokinetic heterogeneity and individualized dosing. Even with lesion-selective enrichment strategies, inter-individual variability in drug absorption, distribution, metabolism, and excretion is likely to be substantial. For inhaled donors, regional deposition depends on airway geometry, ventilation patterns, and the severity of vascular obstruction [45]; patients with advanced disease may have markedly different deposition profiles than those with milder disease. For intravenously administered targeted donors, variability in cardiac output, pulmonary blood flow, and endothelial receptor expression will influence pulmonary homing efficiency. These factors suggest that fixed dosing regimens may be inadequate; future clinical development may require adaptive dosing strategies guided by pharmacokinetic monitoring or biomarker readouts of target engagement. However, the lack of validated real-time H_2_S monitoring tools makes such approaches currently impractical [39,53].

The regulatory pathway for a multifunctional donor system—particularly one involving both a novel chemical entity and a targeting moiety—is likely to be more complex than for a conventional small molecule. In many regulatory frameworks (including FDA and EMA), a conjugate consisting of a small-molecule donor and an antibody or peptide targeting ligand may be classified as a biologic or as a hybrid drug-biologic combination product, depending on the characteristics of the targeting moiety [92]. This classification has profound implications for manufacturing standards, preclinical toxicology requirements, and clinical trial design. Moreover, if the proposed therapeutic strategy involves multiple active components (e.g., a Donor 2.0 plus an HDAC inhibitor), the regulatory pathway becomes even more complex, as each component must be evaluated individually and in combination. Sponsors would need to navigate requirements for drug–drug interaction studies, combination toxicology, and potentially separate Investigational New Drug (IND) applications. These regulatory complexities are not insurmountable, but they require early and ongoing consultation with regulatory agencies—a process that is time-consuming, costly, and subject to considerable uncertainty.

Integration of long-term safety into development planning. While we have discussed specific safety concerns (mitochondrial toxicity, endocrine disruption, HDAC inhibitor toxicities) in Section 6.2, the integration of these considerations into a coherent development plan deserves emphasis. Regulators will expect a systematic, phased approach to safety evaluation: (i) in vitro toxicology screening to identify potential off-target effects and establish preliminary safety margins; (ii) sub-chronic and chronic toxicology studies in relevant animal models (including both rodents and non-rodents where appropriate) to assess cumulative toxicity and identify target organs; (iii) dedicated reproductive and developmental toxicology studies if the target population includes women of childbearing potential; (iv) genotoxicity and carcinogenicity assessments for chronic administration; and (v) rigorous monitoring plans in early-phase clinical trials, including predefined stopping rules. This extensive safety package—which may take several years and substantial financial investment to complete—represents a major practical barrier to the clinical translation of any novel H_2_S donor platform. Moreover, the combination of H_2_S donors with HDAC inhibitors would require even more extensive safety evaluation, as the potential for unexpected synergistic toxicities cannot be predicted from the individual components alone.

The practical challenges outlined above—manufacturing complexity, patient heterogeneity, pharmacokinetic variability, regulatory uncertainty, and the extensive safety evaluation required—collectively represent a formidable barrier to clinical translation. They are not reasons to abandon the proposed strategies, but they are important reminders that the path from conceptual framework to clinical application is long, expensive, and uncertain. We hope that by explicitly acknowledging these challenges, we can encourage the field to address them proactively, rather than viewing them as afterthoughts to be resolved after proof-of-concept has been demonstrated. Future research should include, alongside mechanistic and efficacy studies, systematic attention to manufacturability, patient stratification strategies, pharmacokinetic modeling, and regulatory planning. These efforts are essential to bridge the gap between scientific innovation and clinical impact.

### 6.8. Unresolved Questions and Controversies in the Field

Despite the substantial progress in understanding H_2_S biology and donor design, several fundamental questions and controversies remain unresolved. Acknowledging these uncertainties is essential for guiding future research and for providing readers with a realistic perspective on the state of the field.

Controversy 1: What is the optimal therapeutic strategy—chronic supplementation or episodic reactivation? The field is divided between those who advocate for continuous H_2_S donor administration to maintain sustained plasma levels and those who propose intermittent, “pulse” regimens to mimic the physiological, transient nature of endogenous H_2_S signaling [5,48]. Proponents of continuous administration argue that PAH is a chronic disease requiring persistent H_2_S bioavailability; proponents of episodic reactivation counter that sustained H_2_S elevation may desensitize downstream signaling pathways and increase the risk of toxicity. This debate has not been resolved, and the optimal strategy may differ depending on the specific patient population, disease stage, and donor system used.

Controversy 2: Does H_2_S act primarily as a direct signaling molecule or through secondary mediators (e.g., polysulfides, persulfides, or S-sulfhydration of protein thiols)? The biochemical mechanisms of H_2_S action remain incompletely understood. While S-sulfhydration of proteins (including ETAR and PTEN) has been demonstrated in vitro, the relative contribution of direct H_2_S signaling versus the action of downstream metabolites (such as polysulfides) is unclear [1,31,80]. Some investigators have argued that polysulfides, rather than H_2_S itself, are the primary mediators of many biological effects attributed to H_2_S [69]. If this is correct, then the design of H_2_S donors that preferentially generate specific sulfur species may be more therapeutically relevant than simple H_2_S release. This uncertainty complicates donor design and interpretation of preclinical findings.

Controversy 3: To what extent do preclinical findings in rodent models translate to human PAH? As discussed in Section 6.6, the MCT and Sugen/hypoxia models do not fully recapitulate human PAH. The most obvious limitation is the absence of advanced neointimal and plexiform lesions in standard rodent models [18,37]. However, even within the rodent model literature, there are discrepancies: some studies report substantial hemodynamic benefit from H_2_S donors, while others find only modest effects or significant toxicity at therapeutic doses [35,36]. Whether these discrepancies reflect true biological variability, differences in experimental protocols, or publication bias (favoring positive results) is difficult to determine. This uncertainty underscores the need for independent replication studies and, ultimately, human clinical trials.

Unresolved question 1: What are the optimal biomarkers for patient selection and monitoring? While we have proposed several potential biomarkers (PTEN S-sulfhydration, IP receptor density, ETBR-positive microparticles), none has been validated in prospective clinical studies [90,91]. Plasma H_2_S levels are highly variable and prone to measurement artifacts, making them unsuitable for routine clinical use [53]. The development of reliable, practical biomarkers remains a critical unmet need that will determine whether patient stratification and pharmacodynamic monitoring can be achieved.

Unresolved question 2: What is the safety profile of long-term H_2_S elevation in humans? As discussed in Section 6.2, chronic H_2_S elevation raises concerns about mitochondrial toxicity, endocrine disruption, and central nervous system effects. However, virtually all safety data come from acute exposure studies in animals or from patients with sulfide quinone oxidoreductase deficiency (a rare genetic disorder characterized by chronic H_2_S elevation) [67]. The long-term safety of therapeutic H_2_S elevation in PAH patients is unknown. This uncertainty represents a major barrier to clinical development, as regulators will require substantial safety data before approving chronic H_2_S donor therapy.

Unresolved question 3: How should combination therapy with HDAC inhibitors be optimized? The proposed combination of H_2_S donors with HDAC inhibitors is conceptually appealing but largely unexplored. Questions regarding optimal dosing, scheduling, route of administration, and toxicity profiles remain entirely unanswered. Furthermore, the potential for synergistic toxicity—particularly given that both agents affect cellular redox state and gene expression—has not been systematically evaluated [70]. The combination strategy remains a hypothesis-generating concept, not a validated therapeutic approach.

Summary of unresolved questions. The field of H_2_S donor therapy for PAH is characterized by substantial unresolved questions and controversies. These uncertainties should not be interpreted as reasons to abandon the approach, but rather as important priorities for future research. We encourage investigators to design studies that explicitly address these unanswered questions, rather than assuming that the current mechanistic framework is complete. A more critical, hypothesis-testing approach will accelerate progress and reduce the risk of translational failure.

## 7. Conclusions

This review has systematically articulated a new therapeutic paradigm for PAH centered on H_2_S donors, integrating three major strategic directions that move far beyond the limitations of first-generation compounds. The first direction is Donor 2.0 for precision delivery, which encompasses pathological signal-triggered release (e.g., by ROS, hypoxia, esterases, or MMPs), lesion-selective enrichment via inhalation or active vascular targeting, and organelle-specific localization such as mitochondria-targeted AP39. These features collectively resolve the non-specific toxicity and poor pharmacokinetics that plagued first-generation donors, enabling H_2_S to be released only where and when it is needed. The second direction is synergy between exogenous donors and endogenous activation, where donors provide immediate H_2_S bioavailability as a “bridge” to buy time for epigenetic, oxidative, and metabolic reactivation of the silenced H_2_S-synthesizing enzymes (CSE, CBS, and 3-MST) [55,61]. This integrated approach ultimately reduces dependence on chronic donor administration by restoring the cell’s own capacity for self-regulated H_2_S production. The third direction is repositioning the donor as a systemic sensitizer, shifting its clinical role from a stand-alone vasodilator competitor to a combination therapy enhancer that reverses acquired insensitivity to existing drugs (ERAs, PDE5i, and prostacyclin analogues) [74]. By S-sulfhydrating key signaling nodes such as ETAR/ETBR, PTEN, and contractile proteins, H_2_S donors restore cellular sensitivity and prolong the durable efficacy of standard-of-care regimens.

These three dimensions are not a simple linear progression; rather, they are mutually reinforcing and can be implemented in a stratified, patient-tailored manner. For instance, a patient with newly diagnosed PAH might first receive a pulsed Donor 2.0 regimen to achieve rapid hemodynamic improvement, while simultaneously initiating epigenetic therapy (e.g., low-dose inhaled HDAC inhibitor) to re-awaken endogenous CSE expression [63,71]. Once endogenous H_2_S production is partially restored, the donor can be tapered to a “standby” role, and the patient’s residual sensitivity to standard drugs may be sufficiently enhanced to allow dose reduction of ERAs or PDE5i. This flexible, donor-centered framework aligns with the principles of precision medicine and chronic disease management.

Looking forward, future research should focus on three priority areas. First, the development of hybrid donor molecules that combine pathological-responsive release with intrinsic epigenetic modulating activity—for example, an H_2_S donor conjugated to a histone deacetylase inhibitor or a bromodomain inhibitor, allowing a single molecule to both supply H_2_S and remodel repressive chromatin [38,53,71]. Second, the design and preclinical validation of combination regimens featuring pulsed Donor 2.0 plus intermittent epigenetic modulators, such as weekly inhaled H_2_S microspheres together with bi-weekly nebulized SAHA or entinostat, with pharmacokinetic and pharmacodynamic monitoring to optimize dosing intervals [10,30]. Third, the conduct of sensitizer-oriented clinical trials with therapeutic durability as the primary endpoint, including time to clinical worsening, delay in parenteral prostacyclin escalation, and biomarker-confirmed target re-sensitization (e.g., IP receptor recovery, PTEN S-sulfhydration levels) [81,84,86]. Such trials should enroll patients with documented therapeutic response decay rather than treatment-naïve individuals [80], and they should incorporate patient-reported outcomes to capture the meaningfulness of prolonged stability [88].

In conclusion, the conceptual framework articulated here—from non-specific release to precision delivery, from continuous transfusion to episodic hematopoiesis, and from single-agent competition to systemic sensitization—if validated experimentally through systematic preclinical and clinical investigation, could eventually elevate H_2_S donors from simple chemical releasers to programmable, context-aware, and therapeutically synergistic platforms. At present, however, these strategies remain future research directions rather than technologies approaching clinical application. We emphasize that this framework is a proposal intended to guide future research; its clinical applicability remains to be established through systematic preclinical and clinical investigation [55,61].

Several significant challenges remain. The lack of clinical data for H_2_S donors in PAH, the uncertain safety profile of chronic H_2_S elevation, the absence of validated biomarkers for patient selection and monitoring, and the regulatory complexities of add-on trial designs all require careful attention before clinical translation can be realized. Moreover, the proposed combinatorial and sequential regimens—particularly those involving HDAC inhibitors—will require systematic optimization and rigorous toxicity assessment in preclinical models before any clinical application can be considered. We encourage the field to pursue these investigations with appropriate scientific caution, recognizing both the substantial therapeutic potential and the considerable uncertainties that remain.

This reimagining of H_2_S donor philosophy does not diminish the molecule’s importance; it defines its true role as an indispensable collaborator in the future of PAH therapy. The preliminary tools are beginning to emerge, the biological logic is mechanistically plausible, and the clinical need is urgent. What remains is the systematic generation of preclinical evidence, the establishment of safety profiles, and the collective will to rigorously test these hypotheses through well-designed studies.

## Figures and Tables

**Table 1:** Summary of the three strategic dimensions of the H_2_S donor paradigm shift.

Dimension	Representative Donors/Strategies	Trigger/Mechanism	Therapeutic Goal
Precision Delivery (Donor 2.0)	HSD545 (ROS-responsive) [19]; AP39 (mitochondria-targeted) [11]; ACS14 microspheres (inhalation) [10]	ROS [19], hypoxia [20], esterase [21], MMPs [22]; active vascular targeting [23]; organelle TPP conjugation [11]	Release H_2_S only in diseased pulmonary vasculature; minimize systemic toxicity
Endogenous Synergy	SAHA/TSA (HDACi) [24] + NaHS/GYY4137 [25]; NAC [26] + donor; Sp1/anti-miR-21-5p [27]	Epigenetic derepression (CSE) [24,28]; oxidative reactivation (CBS) [29]; substrate support (3-MST) [30]	Restore self-regulated H_2_S synthesis; reduce dependence on chronic donor administration
Systemic Sensitization	NaHS [31] or GYY4137 [32] combined with ERAs, PDE5i, or prostacyclin analogues	S-sulfhydration of ETAR/ETBR [1], PTEN [31], and contractile proteins; phenotypic redifferentiation [32]	Reverse acquired drug insensitivity; extend durable efficacy of standard-of-care therapies

**Table 2:** Pathological features of the pulmonary arterial hypertension microenvironment and corresponding trigger signals for responsive H_2_S donor design.

Pathological Feature	Trigger Signal	Donor Design Strategy
Oxidative stress	ROS (O_2_^−^, H_2_O_2_) [19]	Thioketal or aryl boronate linkages cleaved by ROS [19,42]
Hypoxia	Low O_2_ tension [20]	Nitroimidazole or azobenzene moieties reductively activated by hypoxia-upregulated reductases [40]
Inflammation	Esterase (highly expressed in activated macrophages) [21]	Esterase-sensitive ester bonds [21,41]
ECM remodeling	MMP-2/9 overexpression [22]	Peptide substrates (e.g., PLGLAG) cleaved by MMP-2/9 [22,43]

**Table 3:** Three silencing mechanisms of endogenous H_2_S-synthesizing enzymes in pulmonary arterial hypertension and their corresponding reactivation strategies.

Enzyme	Silencing Mechanism	Reactivation Strategy	Synergistic Role of Donor
CSE	Chromatin-mediated transcriptional silencing: enrichment of repressive histone mark H3K9me3 and depletion of acetylated H3K9ac at the CTH promoter [28,57,58]	HDAC inhibitors (e.g., SAHA, TSA) [24]; transcriptional co-activators (Sp1) [27]; mRNA stabilizers (anti-miR-21-5p) [27]	Donor provides immediate H_2_S while epigenetic restoration proceeds; reduces inflammation that sustains repressive chromatin [62]
CBS	Oxidative inactivation: peroxynitrite (ONOO^−^) modifies critical cysteine residues in the heme-dependent catalytic domain, disrupting heme coordination [29]	Reducing agents (e.g., DTT) [29]; development of membrane-permeable, non-toxic CBS reactivators	Donor reduces oxidative stress (by scavenging ROS and upregulating SOD), preventing re-inactivation of CBS [62]
3-MST	Substrate starvation: downregulation of CAT limits the production of 3-MP, the preferred substrate for 3-MST [30]	NAC supplementation (increases cysteine and 3-MP) [26]; CAT overexpression (gene therapy) [30]	Donor does not interfere with the cysteine-CAT-3-MP pathway; can be co-administered without competition [21]

**Table 4:** Three distinct mechanisms of acquired therapeutic insensitivity to standard-of-care drugs in pulmonary arterial hypertension and the intervention points of H_2_S donors.

Mechanism of Insensitivity	Description	Intervention by H_2_S Donors
Receptor upregulation and switching	Chronic ETAR blockade by ERAs (bosentan, ambrisentan, macitentan) Compensatory ETBR upregulation on PASMCs (demonstrated in preclinical models) [78] ETBR is poorly antagonized by current ERAs Continues mediating vasoconstriction & proliferation via Gq/11 and β-arrestin	H_2_S S-sulfhydrates conserved cysteines (Cys69 in ETAR; analogous in ETBR) (demonstrated in vitro and in isolated artery preparations) [1] Uncouples both ETAR and ETBR from Gq protein Suppresses downstream ERK1/2 and IP_3_ signaling Silences the entire endothelin axis regardless of subtype abundance (mechanism inferred from in vitro studies)
Bypass pathway activation	Sustained PDE5 inhibition (sildenafil/tadalafil) elevates cGMP Triggers compensatory PI3K/Akt survival-proliferation axis Driven by cGMP-independent feedback loops (ROS, growth factor receptor transactivation) PASMCs bypass the blocked node	H_2_S S-sulfhydrates PTEN at Cys124 (demonstrated in cultured human PASMCs and other cell types) [31] Protects PTEN from oxidative inactivation, preserving its lipid phosphatase activity (in vitro evidence) Active PTEN maintains low PIP_3_ and restrains basal Akt phosphorylation Imposes a vertical brake on PI3K/Akt, acting downstream & independently of cGMP/PKG (evidence from cell culture studies)
Target extinction through phenotypic switch	Prostacyclin analogues (iloprost, treprostinil, epoprostenol) activate IP receptor → ↑cAMP/PKA PASMCs undergo contractile-to-synthetic phenotypic switch (hallmark of PAH remodeling) IP receptor expression declines by up to 70% (observed in cultured PASMCs and animal models) Drug’s molecular target disappears from cell surface	H_2_S promotes PASMC redifferentiation toward mature contractile phenotype (in vitro evidence in human PASMCs) [32] Restores expression of smooth muscle myosin heavy chain, α-SMA Critically, restores IP receptor density (shown in cultured cells) Not direct agonism but state-change therapy: reinstalls the receptor platform for prostacyclin analogues (hypothesis based on in vitro findings)

## Data Availability

No new data were created or analyzed in this study. Data sharing is not applicable to this article.

## Abbreviations

The following abbreviations are used in this manuscript:

3-MP	3-mercaptopyruvate
3-MST	3-mercaptopyruvate sulfurtransferase
6MWD	six-minute walk distance
α-SMA	alpha-smooth muscle actin
BMPR2	bone morphogenetic protein receptor type 2
cAMP	cyclic adenosine monophosphate
CAT	cysteine aminotransferase
CBS	cystathionine β-synthase
cGMP	cyclic guanosine monophosphate
COPD	chronic obstructive pulmonary disease
CSE	cystathionine γ-lyase
CTH	gene encoding CSE
DATS	diallyl trisulfide
DTT	dithiothreitol
EC_50_	half-maximal effective concentration
ECM	extracellular matrix
eNOS	endothelial nitric oxide synthase
ERA	endothelin receptor antagonist
ERK	extracellular signal-regulated kinase
ETAR	endothelin receptor type A
ETBR	endothelin receptor type B
H_2_S	hydrogen sulfide
H3K9ac	acetylated histone H3 lysine 9
H3K9me3	trimethylated histone H3 lysine 9
HDAC	histone deacetylase
HDACi	histone deacetylase inhibitor
IP receptor	prostacyclin receptor
IP_3_	inositol trisphosphate
iPAH	idiopathic pulmonary arterial hypertension
MCT	monocrotaline
MMP	matrix metalloproteinase
mPAP	mean pulmonary arterial pressure
mRNA	messenger RNA
NAC	N-acetylcysteine
NaHS	sodium hydrosulfide
Na_2_S	sodium sulfide
NT-proBNP	N-terminal pro-brain natriuretic peptide
O_2_	oxygen
ONOO^−^	peroxynitrite
PAEC	pulmonary arterial endothelial cell
PAH	pulmonary arterial hypertension
PASMC	pulmonary arterial smooth muscle cell
PDE5	phosphodiesterase-5
PDE5i	phosphodiesterase-5 inhibitor
PECAM-1	platelet endothelial cell adhesion molecule-1
PIP_3_	phosphatidylinositol (3,4,5)-trisphosphate
PKA	protein kinase A
PKG	protein kinase G
PTEN	phosphatase and tensin homolog
PVR	pulmonary vascular resistance
ROS	reactive oxygen species
SAHA	suberoylanilide hydroxamic acid (vorinostat)
siRNA	small interfering RNA
SOD	superoxide dismutase
Sp1	specificity protein 1
TSA	trichostatin A
TTCW	time to clinical worsening
VCAM-1	vascular cell adhesion molecule-1
